# Advanced echocardiographic phenotyping of critically ill patients with coronavirus-19 sepsis: a prospective cohort study

**DOI:** 10.1186/s40560-020-00516-6

**Published:** 2021-01-20

**Authors:** François Bagate, Paul Masi, Thomas d’Humières, Lara Al-Assaad, Laure Abou Chakra, Keyvan Razazi, Nicolas de Prost, Guillaume Carteaux, Genevieve Derumeaux, Armand Mekontso Dessap

**Affiliations:** 1 AP-HP, Hôpitaux universitaires Henri Mondor, Service de Médecine Intensive Réanimation, 94010 Créteil, France; 2 Université Paris Est Créteil, Faculté de Santé de Créteil, IMRB, Groupe de recherche clinique CARMAS, 94010 Créteil, France; 3 AP-HP, Hôpitaux universitaires Henri Mondor, Service de Physiologie, 94010 Créteil, France; 4 INSERM IMRB U955, Université Paris Est Créteil, Créteil, 94010 France

**Keywords:** COVID-19, Sepsis, Cardiac dysfunction, Afterload

## Abstract

**Background:**

Sepsis is characterized by various hemodynamic alterations which could happen concomitantly in the heart, pulmonary and systemic circulations. A comprehensive demonstration of their interactions in the clinical setting of COVID-19 sepsis is lacking. This study aimed at evaluating the feasibility, clinical implications, and physiological coherence of the various indices of hemodynamic function and acute myocardial injury (AMI) in COVID-19 sepsis.

**Methods:**

Hemodynamic and echocardiographic data of septic critically ill COVID-19 patients were prospectively recorded. A dozen hemodynamic indices exploring contractility and loading conditions were assessed. Several cardiac biomarkers were measured, and AMI was considered if serum concentration of high-sensitive troponin T (hs-TNT) was above the 99th percentile, upper reference.

**Results:**

Sixty-seven patients were assessed (55 males), with a median age of 61 [50–70] years. Overall, the feasibility of echocardiographic parameters was very good, ranging from 93 to 100%. Hierarchical clustering method identified four coherent clusters involving cardiac preload, left ventricle (LV) contractility, LV afterload, and right ventricle (RV) function. LV contractility indices were not associated with preload indices, but some of them were positively correlated with RV function parameters and negatively correlated with a single LV afterload parameter. In most cases (*n* = 36, 54%), echocardiography results prompted therapeutic changes. Mortality was not influenced by the echocardiographic variables in multivariable analysis. Cardiac biomarkers’ concentrations were most often increased with high incidence of AMI reaching 72%. hs-TNT was associated with mortality and inversely correlated with most of LV and RV contractility indices.

**Conclusions:**

In this comprehensive hemodynamic evaluation in critically ill COVID-19 septic patients, we identified four homogeneous and coherent clusters with a good feasibility. AMI was common and associated with alteration of LV and RV functions. Echocardiographic assessment had a clinical impact on patient management in most cases.

**Supplementary Information:**

The online version contains supplementary material available at 10.1186/s40560-020-00516-6.

## Background

The pandemic of coronavirus disease 2019 (COVID-19) caused by the novel severe acute respiratory syndrome coronavirus 2 (SARS-CoV-2) represents the greatest global public health crisis of the last decades. Approximatively, up to 40% of hospitalized patients develop severe pneumonia with sepsis requiring intensive care unit (ICU) admission [1]. Sepsis is characterized by various hemodynamic alterations like hypovolemia, systolic and diastolic cardiac dysfunction, and vasoplegia [2]. In sepsis of pulmonary origin (as it is the case with COVID-19), pulmonary circulatory dysfunction (PCD) and mechanical ventilation may favor right ventricle (RV) dysfunction and acute cor pulmonale (ACP) [3]. Multiple alterations may occur in the same patient and give rise to complex interactions. For instance, the function of one ventricle may influence that of the other via the ventricular interdependence phenomenon [4]. Additionally, the loading conditions may affect left ventricle (LV) systolic [2, 5] and diastolic [6] functions. However, a clinical comprehensive demonstration of these interactions in COVID-19 patients is missing.

Systemic [7] and pulmonary [8] vascular endothelial cell infection could accentuate the effect of loading disturbances in COVID-19. Direct cardiac involvement (e.g., myocarditis) has been suggested as a particular feature of COVID-19 [9]. However, the physiological markers of myocardial injury in COVID-19 have been poorly studied. In recent years, new or revisited echocardiographic tools (speckle tracking and tissue Doppler imaging) and parameters (LV end-systolic maximal elastance and ventricular-arterial coupling) have been used to scrutinize cardiac functions [2]. The feasibility, clinical significance, and physiological coherence of these different indices have not been assessed in COVID-19 sepsis.

The aims of the present study were as follows: (i) first, to assess, in COVID-19 patients, the feasibility, clinical implications, and physiological coherence of the various indices of cardiac function in evaluating hemodynamics in sepsis; (ii) second, to assess the physiological correlates of acute myocardial injury in COVID-19 sepsis.

## Methods

### Patients

We conducted a prospective observational monocenter study on all patients diagnosed with RT-PCR-confirmed SARS-CoV-2 sepsis (Sepsis 3 definition [10]) and consecutively admitted to the medical ICU of Henri Mondor Hospital, Créteil, France, between March 8 and April 26, 2020.

The study was approved by the institutional ethical committee of the French Intensive Care Society as a component of standard care, and patient consent was waived. Written and oral information about the study was given to the families. Case severity was evaluated by Simplified Acute Physiology Score II (SAPS II) for acute illness at ICU admission and by Sequential Organ Failure Assessment (SOFA) score for organ dysfunction in sepsis. For patients in shock, norepinephrine was given as the first-choice vasopressor therapy (targeting a mean arterial pressure of 65 mmHg or more). The patients were followed up until day 28.

### Acute myocardial injury

Acute myocardial injury (AMI) was diagnosed if serum concentration of high-sensitive troponin T (hs-TNT) surpassed the 99th percentile, upper reference level [11, 12], i.e., hs-TNT > 14 ng/L. We also measured blood concentrations of N-terminal pro b-type natriuretic peptide (NT-proBNP) and creatinine phosphokinase (CPK). Serum hs-TNT was measured using electrochemiluminescence immunoassays (Cobas e, Roche Diagnostics GmbH, Mannheim, Germany). Serum NT-proBNP levels were measured by electrochemiluminescence (Cobas 6000, Roche Diagnostics GmbH, Mannheim, Germany), and creatinine phosphokinase by Roche/Hitachi Cobas C systems (Roche Diagnostics GmbH, Mannheim, Germany).

### Echocardiography

To evaluate cardiac function, transthoracic echocardiography (TTE) was performed within 72 h of ICU admission by trained operators (competent in advanced critical care echocardiography) using an S7 or E9 ultrasound system (GEMS, Buc, France). All echocardiography tests were carried out in strict respect to current guidelines for care of COVID-19 patients [13]. We used a standard procedure to assess LV and RV sizes, filling, function, and output, as detailed in Additional file 1. All measurements taken for each examination were averaged over a minimum of three cardiac cycles (five to ten in case of non-sinus rhythm). LV hypertrophy and dilatation were defined as per the 2015 update of the American Society of Echocardiography, and the European Association of Cardiovascular Imaging guidelines, using ventricular mass and volume, respectively [14]. Apical long-axis (four- and two-chamber) clips obtained with a frame rate ≥ 50 Hz underwent on-line speckle tracking analyses conducted by two trained operators on the semi-automated EchoPAC package (GEMS, Buc, France) (see Additional file 1).

### Assessment of contractility and loading conditions

LV preload was assessed using the maximal diameter of inferior vena cava (IVC) and estimates of LV filling pressures (E/A and E/e′ ratios) on pulsed-wave Doppler and tissue Doppler upon measuring, for the former early (E) and late (A), and for the latter early (e′) diastolic wave velocity at the lateral mitral valve annulus. LV afterload was assessed using invasively measured diastolic arterial pressure (DAP), systemic vascular resistance (SVR), and end-systolic arterial elastance (AE) (see Additional file 1 for more detail). LV systolic function assessment was based on the following indices: LV ejection fraction (LVEF) obtained by two-dimensional echocardiography, peak systolic wave at the lateral mitral valve annulus (sm) obtained by tissue Doppler imaging [10], LV global longitudinal peak systolic strain (AS) by speckle tracking imaging, LV end-systolic maximal elastance (ME, see ES), and ventricular-arterial coupling (VAC, see ES), which is the ratio of LV end-systolic maximal elastance and end-systolic arterial elastance as previously described [2]. RV systolic function assessment relied on tricuspid annulus plane systolic excursion (TAPSE) and on tissue Doppler peak systolic wave at tricuspid lateral annulus (st). Patients were also stratified into three groups based on the degree of PCD, as previously described [3] and defined by the following: no dysfunction (group 0: pulmonary artery systolic pressure ≤ 40 mmHg with normal RV size and normal interventricular septum kinetics), moderate dysfunction (group 1: pulmonary artery systolic pressure > 40 mmHg or having dilated RV or paradoxical motion of interventricular septum, but without cor pulmonale), and severe dysfunction (group 2: cor pulmonale, defined as septal dyskinesia with dilated RV (end-diastolic RV/LV surface ratio > 0.6)).

### Statistical analysis

Data were analyzed using IBM SPSS Statistics for Windows (version 24.0, IBM Corp, Armonk, NY) and R 3.2.5 (The R Foundation for Statistical Computing, Vienna, Austria). Continuous data were expressed as medians [25th–75th centiles] unless otherwise specified and were compared using the Mann-Whitney test. Categorical variables, expressed as percentages, were evaluated using the chi-square or Fisher exact test. The two aims of our study were set primarily to test the feasibility of various indices of cardiac function, and to assess their physiological coherence using hierarchical clustering; this method builds homogeneous clusters based on dissimilarities or distances between cases, then proceeds iteratively to join the most similar cases. Within the same objective, we analyzed the impact of loading conditions on cardiac contractility by using bivariate correlations that were further summarized in a correlation matrix (corrplot package within R environment). Correlations were tested using Spearman’s method with Benjamini-Hochberg correction to control the false discovery rate at 0.05 level.

Secondly, we built a focused principal component analysis (FPCA; “psy” package, R 3.2.2, the R Foundation for Statistical Computing, Vienna, Austria) based on AMI as the dependent variable with simple graphical display of correlation structures of echocardiographic variables. Two-tailed *p* values < 0.05 were considered significant.

## Results

### Patients’ characteristics, feasibility, and safety of echocardiography

**Fig. 1:**
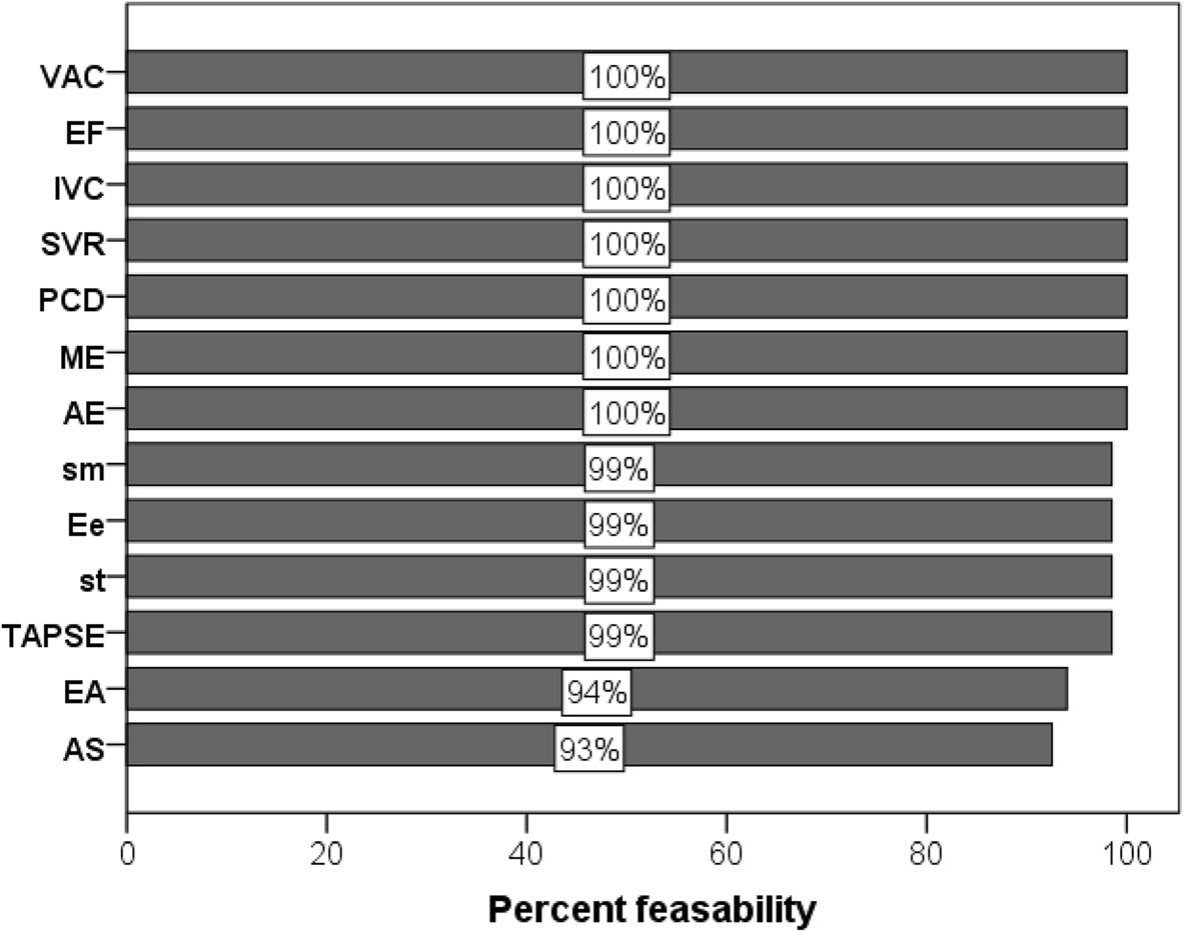
Feasibility of measuring echocardiographic parameters in critically ill patients with coronavirus-19 sepsis. VAC, ventricular-arterial coupling; EF, LV ejection fraction in %; IVC, maximal diameter of inferior vena cava in mm; SVR, systemic vascular resistance in mmHg L^−1^ min; ME, LV end-systolic maximal elastance in mmHg mL^−1^; AE, end-systolic arterial elastance in mmHg mL^−1^; sm, tissue Doppler peak systolic wave at lateral mitral annulus in cm s^−1^; Ee, ratio of early pulsed-wave Doppler to early tissue Doppler diastolic wave velocity at the lateral mitral valve annulus; st, tissue Doppler peak systolic wave at tricuspid lateral annulus in cm s^−1^; TAPSE, tricuspid annulus plane systolic excursion in mm; EA, ratio of early to late diastolic wave velocity at the mitral valve; AS, absolute values of global LV longitudinal peak systolic strain in %; PCD, pulmonary circulatory dysfunction

Patients’ characteristics are provided in Table 1. The study enrolled 67 patients (55 men and 12 women) of a median age of 61 [50–70] years. Most patients were males (*n* = 55, 82%) with cardiovascular risk factors like hypertension (*n* = 36, 54%) and diabetes (*n* = 24, 36%). Many patients were on long-term cardiovascular medications before hospital admission, including beta-blockers (*n* = 15, 22%), statins (*n* = 14, 21%), aspirin (*n* = 13, 19%), angiotensin-converting enzyme inhibitors (*n* = 13, 19%), angiotensin 2 receptor antagonists (*n* = 11, 16%), and furosemide (*n* = 6, 9%). Echocardiography examinations were performed a median of 1 [1–3] day after ICU admission and lasted a median of 21 [15–24] min/exam (not counting preparation and cleaning time). When echocardiography was ordered, 60 (90%) patients required vasopressor infusion and 66 (99%) had invasive mechanical ventilation. Overall, the feasibility of measuring echocardiographic parameters was very good, ranging from 93 to 100% (Fig. 1). Concerning the five sonographers who conducted echocardiography, none were infected by SARS-CoV-2, and their serum serological tests for COVID-19 were all negative 1 month after the end of the study.

**Table 1:** Baseline characteristics and organ failure at time of echocardiography of critically ill patients with coronavirus-19 sepsis, according to survival at day 28

	All patients (***n*** = 67)	Survivors (***n*** = 41)	Non-survivors (***n*** = 26)	***p*** value
**Clinical characteristics and comorbidities**				
Age (years)	61 (50–70)	56 (48–66)	67 (59–72)	< 0.01
Male gender, *n* (%)	55 (82.1%)	33 (80.5%)	22 (84.6%)	0.75
Body mass index (kg/m^2^)	27.3 (24.2–31.9)	27.3 (23.9–31.6)	27.4 (24.6–32.3)	0.70
SAPS II at ICU admission	36 (28–45)	30 (27–40)	41 (36–52)	< 0.01
Diabetes mellitus	24 (36%)	11 (27%)	13 (50%)	0.054
Atrial fibrillation	6 (9%)	3 (7%)	7 (12%)	0.67
Arterial hypertension	36 (54%)	17 (42%)	19 (73%)	0.01
Chronic systolic heart failure	7 (10%)	1 (2%)	6 (23%)	< 0.01
Chronic renal replacement therapy	2 (3%)	1 (2%)	1 (4%)	> 0.99
**Chronic treatments**				
Aspirin	13 (19%)	4 (10%)	9 (35%)	0.02
Anticoagulants	4 (6%)	2 (5%)	2 (8%)	0.64
Statin	14 (21%)	5 (12%)	9 (35%)	0.03
Beta-blockers	15 (22%)	6 (15%)	9 (35%)	0.056
ACE inhibitors or ARB	24 (36%)	9 (22%)	15 (56%)	< 0.01
Mineralocorticoid receptor antagonist	1 (2%)	0	1 (4%)	0.39
Diuretic	6 (9%)	1 (2%)	5 (19%)	0.03
**Organ failure and hemodynamics at time of echocardiography**				
GCS before intubation	15 (15–15)	15 (15–15)	15 (15–15)	0.37
pH	7.36 (7.32–7.42)	7.40 (7.35–7.45)	7.32 (7.23–7.37)	< 0.01
Bicarbonates (mmol/L)	25.8 (22.8–27.0)	26.4 (25.2–28.0)	23.3 (20.4–25.9)	< 0.01
Arterial blood lactate (mmol/L)	1.5 (1.2–2.0)	1.4 (1.2–1.8)	2.0 (1.2–2.6)	0.01
hs-TNT (ng/L)	33 (14–77)	25 (14–44)	52 (23–358)	0.01
Acute myocardial injury*	47 (72%)	26 (67%)	21 (81%)	0.21
CPK (UI/L)	171 (85–364)	129 (63–305)	193 (147–421)	0.03
NT-proBNP (ng/L)	405 (141–1831)	392 (128–1058)	611 (167–3118)	0.18
Creatinine (μmol/L)	103 (72–201)	81 (67–121)	166 (95–256)	< 0.01
Platelet count (G/L)	250 (174–313)	268 (173–330)	229 (181–289)	0.36
Bilirubin (μmol/L)	9 (6–22)	10 (6–28)	9 (6–18)	0.53
SOFA score	8 (6–9)	7 (6–9)	9 (7–10)	0.03
PaO_2_/FiO_2_	139 (105–203)	145 (118–209)	133 (94–197)	0.27
PaCO_2_ (mmHg)	42 (38–47)	42 (39–46)	42 (37–47)	0.38
Invasive mechanical ventilation	66 (99%)	40 (98%)	26 (100%)	> 0.99
PEEP (cmH_2_O)	11 (8–12)	11 (8–12)	11 (9–13)	0.43
Driving pressure	12 (11–15)	13 (10–16)	12 (11–14)	0.63
Crs (mL/cmH_2_O)	32.7 (26.7–41.2)	32.7 (25.7–41.3)	32.3 (26.7–39.2)	0.97
24-h fluid balance (mL)	500 (250–1000)	500 (63–688)	750 (500–1125)	0.03
MAP	78 (71–85)	80 (72–85)	75 (70–86)	0.46
Shock (need for vasopressor)	60 (90%)	36 (88%)	24 (92%)	0.70
Norepinephrine dose (mg/L)	0.6 (0.3–1.7)	0.5 (0.1–1.0)	1.4 (0.5–2.7)	< 0.01
ECMO (%)				0.44
Veno-venous	3 (5%)	2 (5%)	1 (4%)	
Veno-arterial	0	0	0	
Veno-arterio-venous	1 (2%)	0	1 (4%)	
Renal replacement therapy	3 (5%)	3 (8%)	0	0.27

Survival was assessed at day 28. Values are expressed as median (IQR). *COVID-19* coronavirus disease 2019, *SAPS II* Simplified Acute Physiology Score II, *ICU* intensive care unit, *ACE* angiotensin-converting enzyme, *ARB* angiotensin receptor blockers, *GCS* Glasgow coma scale, *hs-TNT* high-sensitive troponin, *CPK* creatinine phosphokinase, *NT-proBNP* N-terminal pro b-type natriuretic peptide, *SOFA* Sequential Organ Failure Assessment, *PaO*_*2*_ partial pressure of oxygen in arterial blood, *PaCO*_*2*_ partial pressure of carbon dioxide in arterial blood, *FiO*_*2*_ fraction of inspired oxygen, *PEEP* positive end-expiratory pressure, *Crs* respiratory system compliance, *MAP* mean arterial pressure, *ECMO* extracorporeal membrane oxygenation

*Acute myocardial injury was assessed in 65 patients with available hs-TNT

### Echocardiographic phenotyping

**Fig. 2:**
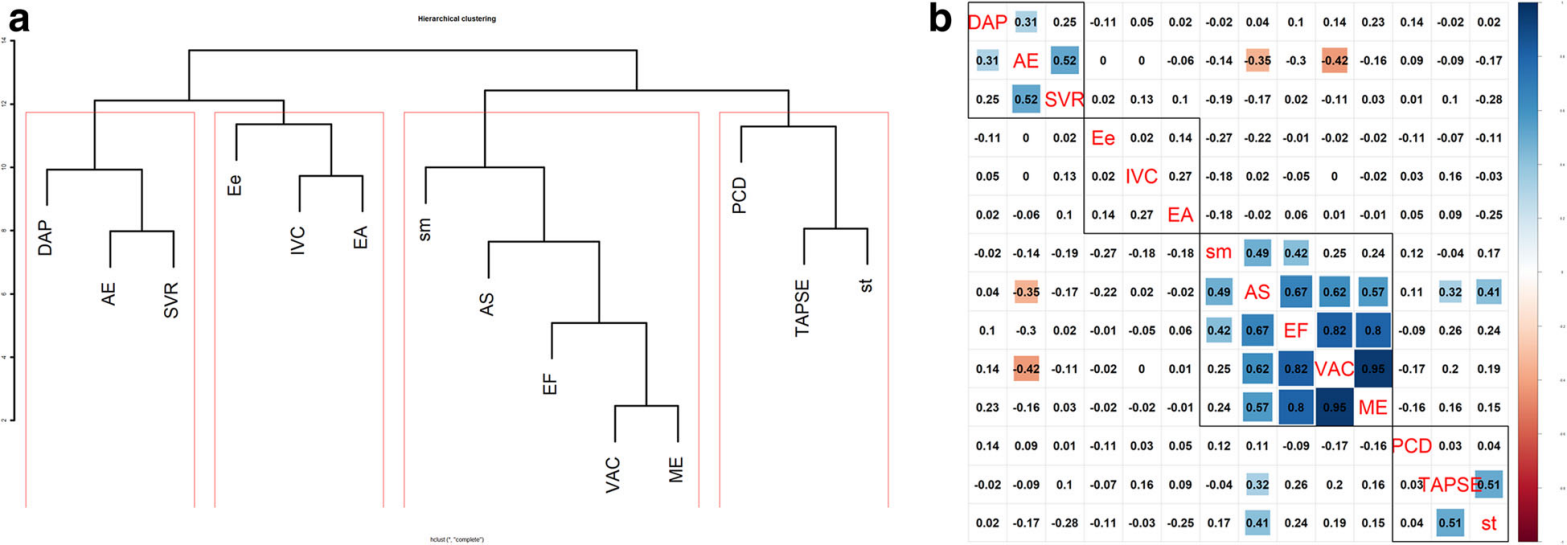
Hierarchical clustering (**a**) and matrix correlation (**b**) of contractility and loading condition indices in critically ill patients with coronavirus-19 sepsis. In **a**, the parameters were reordered using computerized hierarchical clustering with the corrplot package of R statistical environment. Hierarchical clustering is a statistical method for finding comparatively homogeneous clusters of cases based on measured characteristics. The analysis starts with each case as a separate cluster (i.e., there are as many clusters as cases), and then combines the clusters sequentially, reducing the number of clusters at each step. The clustering method uses the dissimilarities between objects. The algorithm uses a set of dissimilarities or distances between cases when constructing the clusters and proceeds iteratively to join the most similar cases. Distances between clusters were recomputed by the Lance-Williams dissimilarity update formula according to the complete linkage method. In **b**, the four big squares drawn in the chart are based on the results of hierarchical clustering and each contains the members of a cluster (LV afterload cluster in the upper-left corner, cardiac preload cluster in the middle upper-left, LV contractility cluster in the middle lower-right, and RV function cluster in the lower-right corner). Numbers and the blue-white-red color spectrum denote the Spearman correlation coefficients (with Benjamini-Hochberg correction to control the false discovery rate at 0.05 level); positive correlations are represented in a blue scale; negative correlations are in a red scale. The surface areas of the colored pixels and their color intensity show the absolute value of corresponding correlation coefficients; non-significant coefficients are left blank. There was a strong correlation between most indices within the LV contractility cluster (blue pixels in the middle lower-right cluster) and within the LV afterload cluster (blue pixels in the upper-left cluster). In addition, some LV contractility indices were negatively correlated with an afterload parameter (red pixels above and to the left of the middle lower-right cluster), and positively correlated with RV function indices (blue pixels below and to the right of the middle lower-right cluster), but not with preload indices. IVC, maximal diameter of inferior vena cava in mm; EA, ratio of early to late diastolic wave velocity at the mitral valve; Ee, ratio of early pulsed-wave Doppler to early tissue Doppler diastolic wave velocity at the lateral mitral valve annulus; EF, LV ejection fraction in %; AS, absolute values of global LV longitudinal peak systolic strain in %; sm, tissue Doppler peak systolic wave at lateral mitral annulus in cm s^−1^; VAC, ventricular-arterial coupling; ME, LV end-systolic maximal elastance in mmHg mL^−1^; AE, end-systolic arterial elastance in mmHg mL^−1^; SVR, systemic vascular resistance in mmHg L^−1^ min; DAP, diastolic arterial pressure in mmHg; PCD, pulmonary circulatory dysfunction; TAPSE, tricuspid annulus plane systolic excursion in mm; st, tissue Doppler peak systolic wave at tricuspid lateral annulus in cm s^−1^

Twenty-one (31%) patients had at least one feature of structural cardiopathy. Briefly, seven had chronic LV hypokinesia (LVEF < 45%), ten had dilated LV, seven had concentric hypertrophy, one had eccentric hypertrophy, and one had significant heart valve disease. In addition, chronic arterial hypertension and chronic atrial fibrillation were present in 36 (54%) and 6 (9%) patients, respectively. Hierarchical clustering of echocardiographic parameters identified four coherent clusters covering the following physiological pathways: LV contractility, RV function, LV afterload, and cardiac preload (Fig. 2a). In the correlation matrix (Fig. 2b), some LV contractility indices were positively correlated with RV function parameters and negatively correlated with a single LV afterload parameter, while no association with cardiac preload was found.

### Clinical implications and outcomes

The echocardiographic assessment modified our therapeutic approach as follows: optimized ventilator settings when patent foramen ovale or acute cor pulmonale were detected, by testing a decrease in PEEP for instance; fluid challenge in patients with preload responsiveness; inotropes in patients with systolic cardiac dysfunction associated with signs of organ hypoperfusion; and anticoagulant therapy in patients with acute cor pulmonale and a high suspicion of pulmonary embolism. Most of the echocardiography results (*n* = 36, 54%) prompted therapeutic changes concerning ventilator settings (*n* = 12, 19%) or medication [in terms of fluids (*n* = 26, 41%), inotropes (*n* = 9, 14%), diuretics (*n* = 2, 3%), anticoagulants, or thrombolytics (*n* = 3, 5%)]. Clinical and biological factors associated with 28-day mortality included older age, high SAPS II score, past medical history of arterial hypertension or chronic systolic heart failure, chronic use of some cardiovascular medications (aspirin, statin, diuretics, angiotensin-converting enzyme inhibitor, or angiotensin receptor blockers), high SOFA score and high doses of norepinephrine at time of echocardiography, lactic acidosis, and increased serum creatinine, hs-TNT, and CPK (Table 1). Mortality at day-28 was not influenced by the echocardiographic variables, except for lower values of E/A in non-survivors as compared with survivors (Table 2, Figure S1). This association between E/A and mortality did not persist in the logistic regression after adjustment on age (OR 0.22, 95% confidence interval 0.03–1.13, *p* = 0.07, Table S3).

**Table 2:** Echocardiographic parameters in critically ill patients with coronavirus-19 sepsis according to survival at day 28

	All patients (***n*** = 67)	Survivors (***n*** = 41)	Non-survivors (***n*** = 26)	***p*** value
***Preload***				
Maximal IVC diameter (mm)	22 (18–25)	23 (19–26)	21 (16–26)	0.36
E/A ratio at mitral valve	1.0 (0.8–1.3)	1.1 (0.9–1.3)	0.8 (0.6–1.1)	< 0.01
E/e′ ratio at mitral valve	7 (6–9)	7 (5–8)	7 (6–10)	0.18
***LV contractility***				
LVEF (%)	60 (49–67)	61 (52–69)	54 (42–67)	0.30
AS (%)	14.8 (17.9–10.2)	15.6 (10.9–18.4)	12.1 (9.1–16.7)	0.12
sm (cm s^−1^)	11 (9–13)	11 (10–16)	11 (10–16)	0.29
VAC	1.9 (1.0–3.0)	2.0 (1.5–2.9)	1.3 (0.8–3.4)	0.17
ME (mmHg mL^−1^)	3.6 (1.8–5.0)	3.9 (2.5–4.7)	2.7 (1.5–6.1)	0.36
***LV afterload***				
AE (mmHg mL^−1^ μg^−1^ kg min)	1.8 (1.6–2.1)	1.7 (1.6–2.0)	1.8 (1.6–2.2)	0.30
SVR (mmHg L^−1^ min)	1096 (908–1245)	1102 (921–1384)	1087 (899–1227)	0.42
DAP (mmHg)	57 (53–66)	57 (55–66)	56 (52–67)	0.34
***RV function***				
TAPSE (mm)	21 (18–25)	22 (20–25)	19 (17–24)	0.11
st (cm/s)	13 (11–17)	13 (11–17)	14 (11–16)	0.98
PCD				0.31
0	28 (42%)	15 (37%)	13 (50%)	
1	8 (12%)	4 (10%)	4 (15%)	
2	31 (46%)	22 (54%)	9 (35%)	
***Global function***				
VTI LVOT (cm)	18 (15–21)	18 (15–21)	18 (15–20)	0.63
Systolic ejection volume (mL)	69 (54–83)	71 (60–85)	68 (53–81)	0.54
Heart rate (rpm)	87 (73–100)	84 (69–97)	90 (76–105)	0.09
Cardiac index (L min)	2.9 (2.4–3.5)	3.0 (2.4–3.4)	2.9 (2.4–3.7)	0.83

Survival was assessed at day 28. *COVID-19* coronavirus disease 2019, *IVC* inferior vena cava, *E/A* ratio of early to late pulsed-wave Doppler of diastolic transmitral flow velocity, *E/e*′ ratio of early pulsed-wave Doppler to early tissue Doppler diastolic wave velocity at the lateral mitral valve annulus, *LV* left ventricle, *RV* right ventricle, *LVEF* left ventricular ejection fraction, *AS* absolute value of left ventricular global longitudinal strain, *VAC* ventricular-arterial coupling, *ME* end-systolic maximal elastance, *e*′ early tissue Doppler diastolic wave velocity at the lateral mitral valve annulus, *AE* arterial elastance, *SVR* systemic vascular resistance, *DAP* diastolic arterial pressure, *TAPSE* tricuspid annulus plane systolic excursion, *sm*: peak of systolic mitral annulus velocity (obtained using pulsed tissue Doppler), *st*: peak of systolic tricuspid annulus velocity (obtained using pulsed tissue Doppler), *PCD* pulmonary circulatory dysfunction, *VTI LVOT* velocity-time integral of left ventricular outflow tract

### Echocardiographic markers in acute myocardial injury

**Fig. 3:**
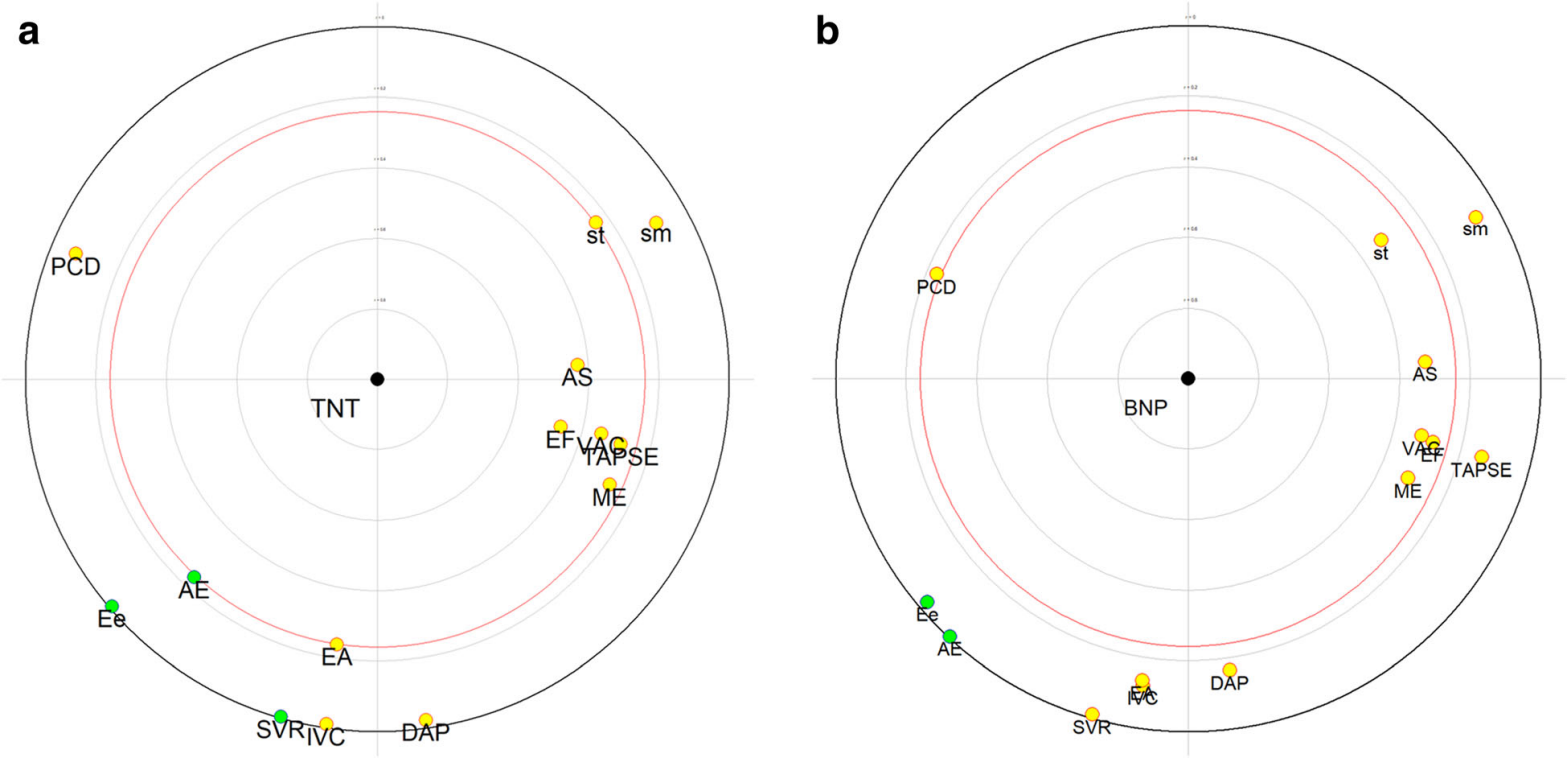
Focused principal component analysis for the association between echocardiographic parameters and cardiac biomarkers: [troponin (**a**) and NT-proBNP (**b**)] in critically ill patients with coronavirus-19 sepsis. Focused principal component analysis (FPCA) is a simple graphical display of correlation structures focusing on a particular dependent variable. The display reflects primarily the correlations between the dependent variable and all other variables (covariates), and secondarily the correlations between the covariates. The dependent variable [high-sensitive troponin T (TNT) in **a** and N-terminal pro b-type natriuretic peptide (BNP) in **b**] is at the center of the each diagram, and the distance of this point to a covariate faithfully represents their pairwise Spearman correlation coefficient (using ranked values of continuous variables). Variables positively and negatively correlated with each dependent variable (TNT and BNP) are in green and yellow, respectively. Covariates significantly correlated with the dependent variable (i.e., *p* value < 0.05) are inside the red circle. The diagram also shows relationships between covariates as follows: correlated covariates are close (for positive correlations, allowing identification of clusters) or diametrically opposite vis-à-vis the origin (for negative correlations), whereas independent covariates form a right angle with the origin. IVC, maximal diameter of inferior vena cava in mm; EA, ratio of early to late diastolic wave velocities at the mitral valve; Ee, ratio of early pulsed-wave Doppler to early tissue Doppler diastolic wave velocity at the lateral mitral valve annulus; EF, LV ejection fraction in %; AS, absolute values of global LV longitudinal peak systolic strain in %; sm, tissue Doppler peak systolic wave at lateral mitral annulus in cm s^−1^; VAC, ventricular-arterial coupling; ME, LV end-systolic maximal elastance in mmHg mL^−1^; AE, end-systolic arterial elastance in mmHg mL^−1^; SVR, systemic vascular resistance in mmHg L^−1^ min; DAP, diastolic arterial pressure in mmHg; PCD, pulmonary circulatory dysfunction; TAPSE, tricuspid annulus plane systolic excursion in mm; st, tissue Doppler peak systolic wave at tricuspid lateral annulus in cm s^−1^

Cardiac biomarkers’ concentrations were higher than normal in the majority of patients (Table 1), and AMI was detected in 47 patients (72%). The blood concentrations of hs-TNT (Fig. 3a) and NT-proBNP (Fig. 3b) were inversely correlated with most of LV and RV contractility indices explored by echocardiography.

## Discussion

The main findings of this study were as follows: (i) bedside focused echocardiographic evaluation of critically ill patients with COVID-19 sepsis was feasible, safe, and incurred therapeutic changes in more than half of them; (ii) echocardiographic indices showed physiological coherence in this population where four coherent hemodynamic clusters were identified, of which two were positively correlated (LV contractility and RV function) and two others were inversely correlated (LV contractility and LV afterload); and (iii) AMI was common and inversely correlated with LV and RV contractility parameters.

The high feasibility, safety, and clinical implications of echocardiography in COVID-19 septic patients suggest its usefulness in this setting as in other forms of sepsis.

### Feasibility

The feasibility of most echocardiography parameters, including strain, outweighed that previously reported, by our laboratory and others, in critically ill septic patients few years ago [2, 15]. Although strain measurement is angle-independent, less subjective than other computer-generated measurements, and very sensitive to detect altered contractility, its assessment requires high frame rate and adequate image quality [16]. The rapid technological evolution in echocardiography hardware (e.g., probe quality) and software (e.g., better recognition of LV walls) could explain our results. Furthermore, all echocardiography tests were performed by expert operators, who routinely use advanced echocardiography tools (including strain) in daily practice.

### Safety

In order to minimize the risk of infection spread among healthcare professionals [17], some authors have recommended to keep the use of echocardiography to a minimum in COVID-19 patients [18, 19]. On the other hand, echocardiography could help clinicians manage COVID-19 sepsis-related hemodynamic alterations. Studies have reported that COVID-19 is associated with specific cardiovascular manifestations, like thromboembolic complications [20] and cardiac injury [21]. The duration of examination (median of 21 min, not including installation or disinfection time) was close to that reported in other critically ill patients [22]. None of the operators in our series got infected, which proves that the recommended measures for the protection of healthcare workers taking care of COVID-19 patients are efficient and that most routine investigations can be safely performed.

### Clinical implications

Most of the echocardiography examinations were followed by therapeutic changes, e.g., administering vasopressors, fluid loading, change of respiratory settings, and use of anticoagulant or thrombolysis, an incentive for a wider indication of echocardiography despite the theoretical risk of infection. A recent large global survey found a comparable impact of echocardiography on management of COVID-19 patients [23].

### Physiological coherence

We identified four coherent and homogeneous clusters involving LV contractility, RV function, LV afterload, and cardiac preload. These findings are consistent with results from previous studies highlighting the physiological coherence of echocardiographic parameters in critically ill patients with sepsis and/or acute respiratory distress syndrome [2, 24]. The absence of correlation between contractility and preload indices matches what previous studies found in human sepsis [2] and is coherent with the fact that echocardiography tests were performed in patients after the initiation of resuscitation and receiving variable amounts of fluids. The association between LV contractility and RV function is also compatible with previous reports results and may be explained by the physiological biventricular interdependence [25]. The inverse correlation between LV contractility and LV afterload is consistent with previous reports, highlighting the role of afterload on contractility indices in sepsis [2]. Overall, our results suggest that COVID-19 induces a myriad of hemodynamic alterations similar to those observed in other forms of sepsis.

### Myocardial injury and outcomes

Several studies in COVID-19 with or without critical illness have suggested an association between markers of RV/LV dysfunction and COVID-19 related death [26, 27]. In our work, no association was found between 28-day mortality and echocardiographic variables, except for lower E/A values in non-survivors. This finding may suggest a detrimental role of diastolic function as previously reported in other forms of sepsis [28]. However, this association did not persist after adjustment on age, a well-known strong prognostic factor in COVID-19. E/A is altered with aging and in case of chronic arterial hypertension [29], two common features in severe forms of COVID-19. As expected and demonstrated in other studies [9, 30], acute myocardial damage was associated with mortality. The prevalence of AMI in our cohort was similar to that in a larger echocardiographic study [30]. Many reports have documented direct myocardial injury incurred by coronavirus-19 [31]. The association between myocardial injury and indices of LV and RV function in our study is in accordance with this hypothesis.

### Strengths and limitations

The strengths of our study rely on the detailed phenotyping using advanced echocardiography, and the exploration of a majority of patients with critical COVID-19, unlike previous studies on the topic [23, 32]. Our study has some limitations. First, the design was monocentric with a limited number of patients (*n* = 67); for this reason, we were not able to carry out robust multivariable analyses and could only cluster echo-parameters, but not patient groups. Some patients were assessed under ECMO, which may alter hemodynamics, but we aimed at exploring all septic critically ill COVID-19 patients, including the most severe. Another important limitation is that transthoracic (TTE) rather than transesophageal echocardiography (TEE) was used, knowing that the latter may outperform TTE for some explorations [33]. The choice of routine TTE was imposed by the fear of specific contamination risks associated with esophageal intubation [34] and by the constraints related to probe sterilization in the context of pandemics. Whether the routine use of TEE may have a similar safety warrants further research. Third, not all patients in the cohort could be assessed at the very early stage. Indeed, our ICU is a tertiary center, and some patients were referred from another ICU. Finally, due to the workload, we were unable to record serial echocardiographic examinations.

## Conclusion

Hereby, we report a comprehensive hemodynamic evaluation by echocardiography in a cohort of patients with COVID-19 sepsis. We identified four homogeneous and coherent physiological clusters. Indices of LV systolic function were positively correlated with RV function and inversely correlated with one LV afterload parameter, while no association was found with cardiac preload. AMI was common and associated with alteration of LV and RV functions. Feasibility and safety of echocardiography were good, and the test had a clinical impact on patient management in a majority of cases.

## Supplementary Information

**Additional file 1.** Complement on methods: echocardiography, Speckle tracking imaging, assessment of contractility and loading conditions and statistical analysis.

**Additional file 2: Fig. S1.** Forest plot of odds ratios (with 95% confidence interval) of variables associated with day-28 mortality by univariate logistic regression.

**Additional file 3: Table S1.** Baseline characteristics and organ failure at time of echocardiography in critically-ill patients with Coronavirus -19 sepsis, according to the need for vasopressor.

**Additional file 4: Table S2.** Echocardiographic parameters in critically-ill patients with Coronavirus -19 sepsis according to the need for vasopressor.

**Additional file 5: Table S3.** Association between E/A and mortality at day-28 by logistic regression after adjustment for age.

## Data Availability

All data generated and analyzed during the study are included in the published article and can be shared upon request. All authors helped to revise the draft of the manuscript. All authors read and approved the final manuscript.

## Abbreviations

COVID-19: Coronavirus disease 2019
SARS-CoV-2: Severe acute respiratory syndrome coronavirus 2
ICU: Intensive care unit
PVD: Pulmonary vascular dysfunction
RV: Right ventricle
ACP: Acute cor pulmonale
LV: Left ventricle
RT-PCR: Real-time reverse transcriptase-polymerase chain reaction
SAPS II: Simplified Acute Physiology Score II
SOFA: Sequential Organ Failure Assessment
hs-TNT: High-sensitive troponin T
NT-proBNP: N-terminal pro b-type natriuretic peptide
CPK: Creatinine phosphokinase
TTE: Transthoracic echocardiography
E/A: Ratio of early to late diastolic wave velocities at the mitral valve
E/e′: Ratio of early pulsed-wave Doppler to early tissue Doppler diastolic wave velocity at the lateral mitral valve annulus
IVC: Inferior vena cava
DAP: Diastolic arterial pressure
SVR: Systemic vascular resistance
AE: End-systolic arterial elastance
LVEF: Left ventricular ejection fraction
sm: Tissue Doppler peak systolic wave at lateral mitral annulus
AS: Global left ventricle longitudinal peak systolic strain
ME: LV end-systolic maximal elastance
VAC: Ventricular-arterial coupling
TAPSE: Tricuspid annulus plane systolic excursion
st: Tissue Doppler peak systolic wave at tricuspid lateral annulus
TEE: Transesophageal echocardiography
