# Supplementary material for: Advanced echocardiographic phenotyping of critically ill patients with coronavirus-19 sepsis: a prospective cohort study
Source: J Intensive Care. 2021 Jan 20;9:12. doi: 10.1186/s40560-020-00516-6 (PMC7816136; doi:10.1186/s40560-020-00516-6)
Supplement: Supplementary file 1 — Additional file 1. Complement on methods: echocardiography, Speckle tracking imaging, assessment of contractility and loading conditions and statistical analysis. [file 40560_2020_516_MOESM1_ESM.doc]

Additional file 1

Advanced echocardiographic phenotyping of critically-ill patients with Coronavirus-19 sepsis: a prospective cohort study

François Bagate, MD;^1,2^ Paul Masi, MD;^1,2^ Thomas d’Humières, MD;^3,4^ Lara Al-Assaad, MD;^3,4^ ; Laure Abou Chakra, MD^3,4^; Keyvan Razazi, MD;^1,2^ Nicolas de Prost MD, PhD;^1,2^  Guillaume Carteaux, MD, PhD^1,2^; Genevieve Derumeaux, MD, PhD;^3,4^ Armand Mekontso Dessap, MD, PhD.^1,2^

**PATIENTS AND METHODS**

**Echocardiography**

To evaluate cardiac function, we conducted transthoracic echocardiography (TTE) within the first three days of ICU admission for COVID-19 sepsis. These echocardiography tests were performed by trained operators (competent in advanced critical care echocardiography) [1] using an S7 or E9 ultrasound system (GEMS, Buc, France) with a standard procedure [2]. Briefly, the following echocardiographic views were examined: four-chamber and two-chamber long-axis views to assess left ventricle (LV) ejection fraction (computed from LV volume using bi-plane Simpson method [3] when image quality was good, or visually estimated when poor image quality did not allow sufficient identification of the endocardium) [4], tissue Doppler peak systolic wave at the lateral mitral valve annulus) [5], right ventricle size (a dilated RV was defined by an end-diastolic RV/LV area ratio >0.6) [6], long-axis M-mode view of the inferior vena cava to assess its size [7], LV filling pressures ⦋using pulsed-wave Doppler early (E) and late (A) diastolic wave velocities at the mitral valve, and tissue Doppler early (e’) diastolic wave velocity at the lateral mitral valve annulus [8]. Pulsed-wave Doppler flows were obtained at the aortic valve to assess aortic velocity-time integral for cardiac output computation. All measures were averaged over a minimum of three cardiac cycles (five to ten in case of non-sinus rhythm).

**Speckle tracking imaging**

Apical long-axis (four- and two-chamber) clips obtained with a frame rate ≥50 Hz underwent online speckle tracking analyses using the semi-automated EchoPAC package (GEMS, Buc, France). The average of three consecutive heart cycles was used to calculate global longitudinal peak systolic strain and strain-rate of the LV.

**Assessment of contractility and loading conditions**

Preload was assessed using estimates of LV filling pressures (E/A and E/e’ ratios) [8] and maximal diameter of inferior vena cava (as surrogates of fluid responsiveness) [7]. Afterload was assessed using the following indices: i) diastolic arterial pressure (which is often used as a surrogate of LV afterload in clinical practice) [9]; ii) systemic vascular resistance (the most commonly used marker of vascular tone) [10]
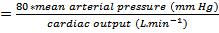
;

iii) end-systolic arterial elastance (to reflect the pulsatile component of peripheral load) [11–13]
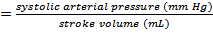
.

LV systolic function was assessed using indices obtained by two-dimensional echocardiography (LVEF), tissue Doppler imaging (tissue Doppler peak systolic wave at the lateral mitral valve annulus), speckle tracking imaging (global longitudinal peak systolic strain of the LV), and two additional indices to reflect net cardiovascular performance and heart-arterial interaction, respectively, as:

1. LV end-systolic maximal elastance
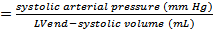
 [14,15], and ii) ventricular-arterial coupling, which is the ratio of LV end-systolic maximal elastance and end-systolic arterial elastance, namely
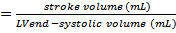
 [11]
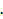
 LV end-systolic volume was obtained from Simpson method [3], and stroke volume was derived from left ventricle outflow track diameter and aortic velocity-time integral.

**Statistical Analysis**

The parameters recorded were the following: maximal size of inferior vena cava (IVC) in mm; ratio of early to late diastolic wave velocities at the mitral valve (EA); ratio of early pulsed-wave Doppler to early tissue Doppler diastolic wave velocity at the lateral mitral valve annulus (Ee); LV ejection fraction in % (LVEF); absolute values of global LV longitudinal peak systolic strain in % (AS);; tissue Doppler peak systolic wave at lateral mitral (sm) and tricuspid (st) annulus in cm.s^-1^; tricuspid annular plane systolic excursion (TAPSE) in mm; ventricular-arterial coupling (VAC); LV end-systolic maximal elastance in mmHg.mL^-1^ (ME); end-systolic arterial elastance in mmHg.mL^-1^ (AE); systemic vascular resistance in mmHg.L^-1^.min (SVR); diastolic arterial pressure in mmHg (DAP).

The parameters were reordered using computerized hierarchical clustering with the hclust and corrplot packages of R statistical environment. Hierarchical clustering is a statistical method to look for comparatively homogeneous clusters of cases based on measured characteristics. The analysis starts with each case as a separate cluster (i.e. there are as many clusters as cases), and then combines the clusters sequentially, reducing the number of clusters at each step. The clustering method uses the dissimilarities between objects. The algorithm uses a set of dissimilarities or distances between cases when constructing the clusters and proceeds iteratively to join the most similar cases. Distances between clusters were recomputed by Lance-Williams dissimilarity update formula according to the complete linkage method. In general, there are many choices of cluster analysis methodology. The hclust function in R uses the complete linkage method for hierarchical clustering by default. This particular clustering method defines the distance between two clusters as the maximum distance between their individual components. At every stage of the clustering process, each two very near clusters are merged into a new cluster. The process is repeated until the whole data set is agglomerated into one single cluster. Complete linkage has a tendency to produce compact bunches. We chose this method to minimize the spread within each cluster [16].

**References**

1. Expert Round Table on Echocardiography in ICU. International consensus statement on training standards for advanced critical care echocardiography. Intensive Care Med. 2014;40:654–66.

2. Vieillard-Baron A, Prin S, Chergui K, Dubourg O, Jardin F. Hemodynamic instability in sepsis: bedside assessment by Doppler echocardiography. Am J Respir Crit Care Med. 2003;168:1270–6.

3. Lang RM, Bierig M, Devereux RB, Flachskampf FA, Foster E, Pellikka PA, et al. Recommendations for chamber quantification: a report from the American Society of Echocardiography’s Guidelines and Standards Committee and the Chamber Quantification Writing Group, developed in conjunction with the European Association of Echocardiography, a branch of the European Society of Cardiology. J Am Soc Echocardiogr Off Publ Am Soc Echocardiogr. 2005;18:1440–63.

4. Gudmundsson P, Rydberg E, Winter R, Willenheimer R. Visually estimated left ventricular ejection fraction by echocardiography is closely correlated with formal quantitative methods. Int J Cardiol. 2005;101:209–12.

5. Seo J-S, Kim D-H, Kim W-J, Song J-M, Kang D-H, Song J-K. Peak systolic velocity of mitral annular longitudinal movement measured by pulsed tissue Doppler imaging as an index of global left ventricular contractility. Am J Physiol Heart Circ Physiol. 2010;298:H1608-1615.

6. Boissier F, Katsahian S, Razazi K, Thille A, Roche-Campo F, Leon R, et al. Prevalence and prognosis of cor pulmonale during protective ventilation for acute respiratory distress syndrome. Intensive Care Med. 2013;39:1725–33.

7. Vieillard-Baron A, Evrard B, Repessé X, Maizel J, Jacob C, Goudelin M, et al. Limited value of end-expiratory inferior vena cava diameter to predict fluid responsiveness impact of intra-abdominal pressure. Intensive Care Med [Internet]. 2018 [cited 2018 Jan 26]; Available from: http://link.springer.com/10.1007/s00134-018-5067-2

8. Nagueh SF. Non-invasive assessment of left ventricular filling pressure: Non-invasive estimation of LV filling pressure. Eur J Heart Fail. 2018;20:38–48.

9. Chirinos JA, Segers P. Noninvasive Evaluation of Left Ventricular Afterload. Hypertension. 2010;56:563–70.

10. Greim CA, Roewer N, Schulte am Esch J. Assessment of changes in left ventricular wall stress from the end-systolic pressure-area product. Br J Anaesth. 1995;75:583–7.

11. Sunagawa K, Maughan WL, Burkhoff D, Sagawa K. Left ventricular interaction with arterial load studied in isolated canine ventricle. Am J Physiol. 1983;245:H773-780.

12. Devereux RB. Toward a more complete understanding of left ventricular afterload. J Am Coll Cardiol. 1991;17:122–4.

13. Sandler H, Dodge HT. LEFT VENTRICULAR TENSION AND STRESS IN MAN. Circ Res. 1963;13:91–104.

14. Bombardini T, Costantino MF, Sicari R, Ciampi Q, Pratali L, Picano E. End-systolic elastance and ventricular-arterial coupling reserve predict cardiac events in patients with negative stress echocardiography. BioMed Res Int. 2013;2013:235194.

15. Sagawa K, Suga H, Shoukas AA, Bakalar KM. End-systolic pressure/volume ratio: a new index of ventricular contractility. Am J Cardiol. 1977;40:748–53.

16. Oksanen J. Cluster analysis: tutorial with R. Univ Oulu Oulu [Internet]. 2010 [cited 2016 Dec 14]; Available from: http://cc.oulu.fi/~jarioksa/opetus/metodi/sessio3res.pdf
