# Supplementary figures and images for: Advanced echocardiographic phenotyping of critically ill patients with coronavirus-19 sepsis: a prospective cohort study
Source: J Intensive Care. 2021 Jan 20;9:12. doi: 10.1186/s40560-020-00516-6 (PMC7816136; doi:10.1186/s40560-020-00516-6)

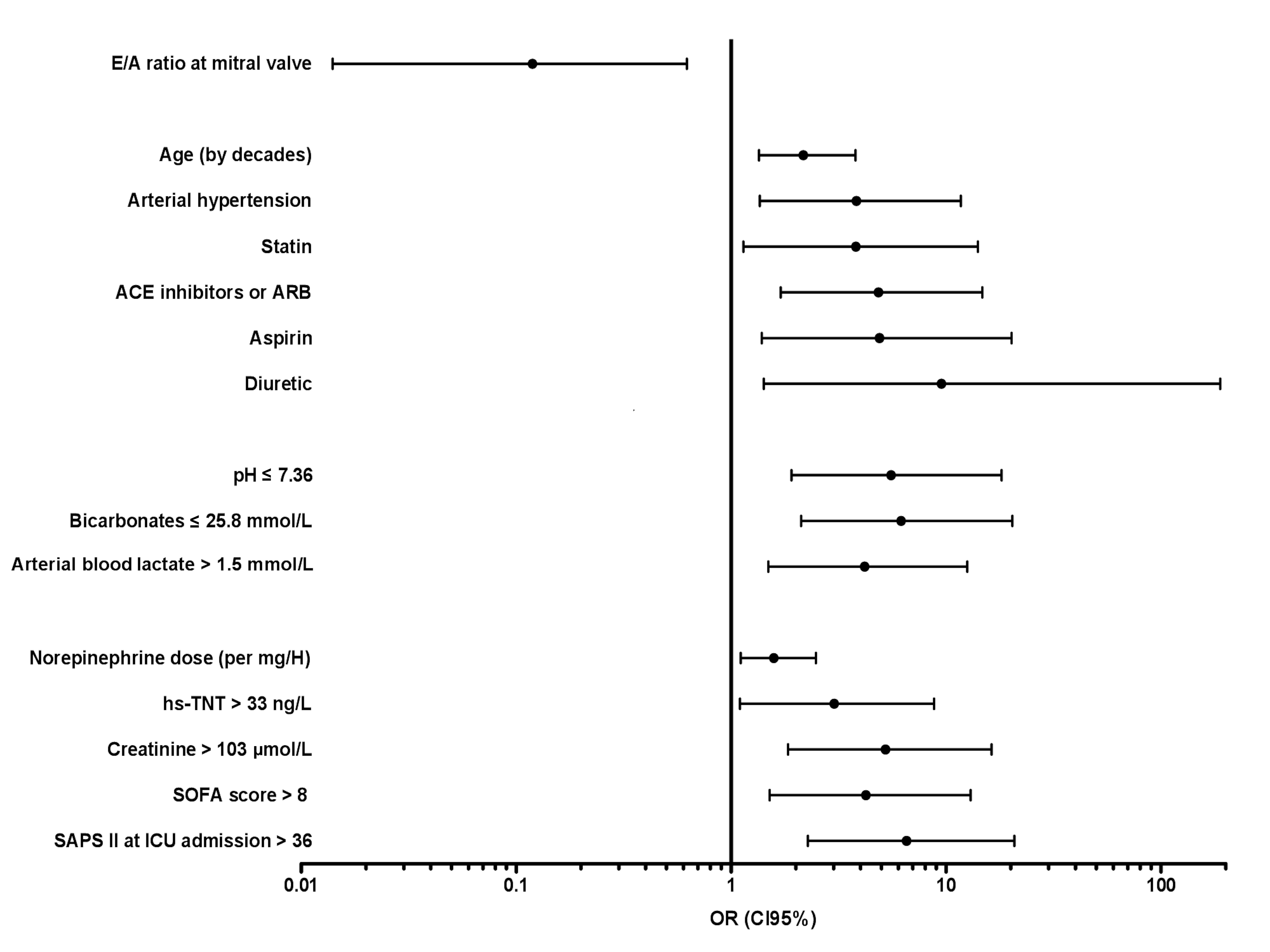

Supplement: Supplementary file 2 — Additional file 2: Fig. S1. Forest plot of odds ratios (with 95% confidence interval) of variables associated with day-28 mortality by univariate logistic regression. [file 40560_2020_516_MOESM2_ESM.tiff]
