# Supplementary material for: Advanced echocardiographic phenotyping of critically ill patients with coronavirus-19 sepsis: a prospective cohort study
Source: J Intensive Care. 2021 Jan 20;9:12. doi: 10.1186/s40560-020-00516-6 (PMC7816136; doi:10.1186/s40560-020-00516-6)
Supplement: Supplementary file 3 — Additional file 3: Table S1. Baseline characteristics and organ failure at time of echocardiography in critically-ill patients with Coronavirus -19 sepsis, according to the need for vasopressor. [file 40560_2020_516_MOESM3_ESM.docx]

| **Table S1** Baseline characteristics and organ failure at time of echocardiography in critically-ill patients with Coronavirus -19 sepsis, according to the need for vasopressor | | | | |
| --- | --- | --- | --- | --- |
|  | **All patients**  **(n=67)** | **Shock**  **(n=60)** | **Without shock**  **(n=7)** | ***P* value** |
| **Clinical characteristics and comorbidities** |  |  |  |  |
| Age (years) | 61 (50-70) | 61 (52-70) | 52 (49-73) | 0.46 |
| Male gender, n (%) | 55 (82.1%) | 49 (81.7%) | 6 (85.7%) | 0.79 |
| Body mass index (Kg/m^2^) | 27.3 (24.2-31.9) | 27.1 (24.2-31.7) | 29.7 (23.0-35.3) | 0.63 |
| SAPS II at ICU admission | 36 (28-45) | 44 (32-50) | 34 (24-44) | 0.15 |
| Diabetes Mellitus | 24 (36%) | 22 (37%) | 2 (29%) | >0.99 |
| Atrial fibrillation | 6 (9%) | 5 (8%) | 1 (14%) | 0.50 |
| Arterial hypertension | 36 (54%) | 33 (55%) | 3 (43%) | 0.70 |
| Chronic systolic heart failure | 7 (10%) | 7 (12%) | 0 | >0.99 |
| Chronic renal replacement therapy | 2 (3%) | 1 (2%) | 1 (14%) | 0.20 |
| **Chronic treatments** |  |  |  |  |
| Aspirin | 13 (19%) | 12 (20%) | 1 (14%) | >0.99 |
| Anticoagulants | 4 (6%) | 4 (7%) | 0 | >0.99 |
| Statin | 14 (21%) | 12 (20%) | 2 (29%) | 0.63 |
| Beta-blockers | 15 (22%) | 12 (20%) | 3 (43%) | 0.18 |
| ACE inhibitors or ARB | 24 (36%) | 23 (38%) | 1 (14%) | 0.41 |
| Mineralocorticoid receptor antagonist | 1 (2%) | 1 (2%) | 0 | >0.99 |
| Diuretic | 6 (9%) | 5 (8%) | 1 (14%) | 0.50 |
| **Organ failure and hemodynamics at time of echocardiography** |  |  |  |  |
| GCS before intubation | 15 (15-15) | 15 (15-15) | 15 (15-15) | 0.97 |
| pH | 7.36 (7.32-7.42) | 7.36 (7.32-7.41) | 7.36 (7.31-7.46) | 0.55 |
| Bicarbonates (mmol/L) | 25.8 (22.8-27.0) | 25.8 (23.0-27.0) | 24.9 (21.1-27.0) | 0.66 |
| Arterial blood lactate (mmol/L) | 1.5 (1.2-2.0) | 1.5 (1.2-2.1) | 1.4 (0.8-1.9) | 0.21 |
| hs-TNT (ng/L) | 33 (14-77) | 35 (14-103) | 25 (7-38) | 0.16 |
| Acute myocardial injury | 47 (72%) | 43 (74%) | 4 (57%) | 0.39 |
| CPK (UI/L) | 171 (85-364) | 181 (104-391) | 58 (40-106) | 0.02 |
| NT-proBNP (ng/L) | 405 (141-1,831) | 510 (153-1,896) | 371 (15-859) | 0.24 |
| Creatinine (μmol/L) | 103 (72-201) | 105 (72-204) | 81 (56-140) | 0.42 |
| Platelet count (G/L) | 250 (174-313) | 247 (174-308) | 311 (170-353) | 0.59 |
| Bilirubin (μmol/L) | 9 (6-22) | 9 (6-26) | 8 (6-10) | 0.16 |
| SOFA score | 8 (6-9) | 8 (7-10) | 4 (3-7) | <0.01 |
| PaO_2_/FiO_2_ | 139 (105-203) | 142 (106-194) | 132 (98-270) | 0.66 |
| PaCO_2_ (mmHg) | 42 (38-47) | 43 (39-47) | 40 (36-42) | 0.13 |
| Invasive mechanical ventilation | 66 (99%) | 59 (98%) | 7 (100%) | >0.99 |
| PEEP (cmH_2_O) | 11 (8-12) | 11 (9-12) | 9 (5-10) | 0.04 |
| Driving pressure | 12 (11-15) | 13 (11-15) | 12 (10-13) | 0.27 |
| Crs (mL/cmH_2_O) | 32.7 (26.7-41.2) | 31.0 (26.7-40.4) | 40.2 (28.6-44.3) | 0.21 |
| 24-hour fluid balance (mL) | 500 (250-1,000) | 500 (250-1,000) | 500 (188-500) | 0.25 |
| MAP (mmHg) | 78 (71-85) | 78 (70-84) | 96 (76-103) | 0.03 |
| Norepinephrine dose (mg/L) | 0.6 (0.3-1.7) | 0.6 (0.3-1.9) | 0 | - |
| ECMO (%) |  |  |  | 0.40 |
| Veno-venous | 3 (5%) | 2 (3%) | 1 (14%) |  |
| Veno-arterial | 0 | 0 | 0 |  |
| Veno-arterio-venous | 1 (2%) | 1 (2%) | 0 |  |
| Renal replacement therapy | 3 (5%) | 3 (5%) | 0 | >0.99 |
| Survival was assessed at day-28. *acute myocardial injury was assessed in 65 patients with available hs-TNT, Values are expressed as median (IQR). COVID-19: coronavirus disease 2019, SAPS II: Simplified Acute Physiology Score II, ICU: intensive care unit, ACE: Angiotensin-converting enzyme, ARB: Angiotensin receptors blockers, GCS: Glasgow coma scale, Hs-troponin: high-sensitive troponin, CPK: creatinine phosphokinase, NT-proBNP: N-terminal pro B-type natriuretic peptide, SOFA: Sequential Organ Failure Assessment, PaO_2_: partial pressure of oxygen in arterial blood; PaO_2_: partial pressure of carbon dioxide in arterial blood;, FiO_2_: fraction of inspired oxygen, PEEP: positive end-expiratory pressure, Crs: respiratory system compliance, MAP: mean arterial pressure, ECMO: extracorporeal membrane oxygenation. | | | | |
