# Supplementary material for: Advanced echocardiographic phenotyping of critically ill patients with coronavirus-19 sepsis: a prospective cohort study
Source: J Intensive Care. 2021 Jan 20;9:12. doi: 10.1186/s40560-020-00516-6 (PMC7816136; doi:10.1186/s40560-020-00516-6)
Supplement: Supplementary file 4 — Additional file 4: Table S2. Echocardiographic parameters in critically-ill patients with Coronavirus -19 sepsis according to the need for vasopressor. [file 40560_2020_516_MOESM4_ESM.docx]

| **Table S2** Echocardiographic parameters in critically-ill patients with Coronavirus -19 sepsis according to the need for vasopressor | | | | |
| --- | --- | --- | --- | --- |
|  | **All patients**  **(n=67)** | **Shock**  **(n=60)** | **Without shock**  **(n=7)** | ***P* value** |
| ***Preload*** |  |  |  |  |
| Maximal IVC diameter (mm) | 22 (18-25) | 6 (0-23) | 0 (0-15) | 0.37 |
| E/A ratio at mitral valve | 1.0 (0.8-1.3) | 1.0 (0.8-1.3) | 0.9 (0.7-1.2) | 0.62 |
| E/e’ ratio at mitral valve | 7 (6-9) | 7 (6-9) | 5 (5-8) | 0.09 |
| ***LV contractility*** |  |  |  |  |
| LVEF (%) | 60 (49-67) | 59 (45-67) | 62 (54-70) | 0.32 |
| AS (%) | 14.8 (17.9-10.2) | 14.5 (10.2-17.5) | 18.5 (12.9-19.4) | 0.16 |
| sm (cm. s^-1^) | 11 (9-13) | 11 (8-13) | 13 (9-15) | 0.49 |
| VAC | 1.9 (1.0-3.0) | 1.8 (1.0-2.9) | 2.5 (1.8-4.2) | 0.20 |
| ME (mmHg.mL^-1^) | 3.6 (1.8-5.0) | 3.2 (1.8-4.7) | 4.5 (3.0-5.7) | 0.23 |
| ***LV afterload*** |  |  |  |  |
| AE (mmHg.mL^-1^.µg^-1^.kg.min) | 1.8 (1.6-2.1) | 1.8 (1.6-2.2) | 1.7 (1.6-1.8) | 0.62 |
| SVR (mmHg.L^-1^.min) | 1,096 (908-1,245) | 1,099 (915-1,250) | 1,077 (904-1,209) | 0.62 |
| DAP (mmHg) | 57 (53-66) | 57 (52-65) | 68 (54-80) | 0.08 |
| ***RV function*** |  |  |  |  |
| TAPSE (mm) | 21 (18-25) | 21 (18-24) | 25 (18-34) | 0.09 |
| st (cm/s) | 13 (11-17) | 13 (11-16) | 19 (13-24) | 0.02 |
| PCD |  |  |  | 0.57 |
| 0 | 28 (42%) | 25 (42%) | 3 (43%) |  |
| 1 | 8 (12%) | 8 (13%) | 0 |  |
| 2 | 31 (46%) | 27 (45%) | 4 (57%) |  |
| ***Global function*** |  |  |  |  |
| VTI LVOT (cm) | 18 (15-21) | 18 (15-21) | 17 (16-25) | 0.64 |
| Systolic ejection volume (mL) | 69 (54-83) | 68 (54-80) | 87 (68-97) | 0.06 |
| Heart rate (rpm) | 87 (73-100) | 88 (73-100) | 87 (73-101) | 0.98 |
| Cardiac Index (L.min) | 2.9 (2.4-3.5) | 2.9 (2.4-3.3) | 3.6 (3.2-3.9) | 0.06 |
| Survival was assessed at day-28. COVID-19: coronavirus disease 2019, IVC: inferior vena cava, E/A: ratio of early to late pulsed-wave Doppler of diastolic transmitral flow velocity, E/e’: ratio of early pulsed-wave Doppler to early tissue Doppler diastolic wave velocity at the lateral mitral valve annulus, LV: left ventricle, RV: right ventricle, LVEF: left ventricular ejection fraction, AS: absolute value of left ventricular global longitudinal strain, VAC: ventricular-arterial coupling, ME: End-systolic maximal elastance, e’: early tissue Doppler diastolic wave velocity at the lateral mitral valve annulus, AE: arterial elastance, SVR: systemic vascular resistance, DAP: diastolic arterial pressure, TAPSE: tricuspid annulus plane systolic excursion, sm: mitral valve: peak of systolic mitral annulus velocity (obtained using pulsed tissue Doppler), st: tricuspid valve: peak of systolic tricuspid annulus velocity (obtained using pulsed tissue Doppler), PCD: pulmonary circulatory dysfunction, VTI LVOT: velocity-time integral of left ventricular outflow tract. | | | | |
