# Supplementary material for: Advanced echocardiographic phenotyping of critically ill patients with coronavirus-19 sepsis: a prospective cohort study
Source: J Intensive Care. 2021 Jan 20;9:12. doi: 10.1186/s40560-020-00516-6 (PMC7816136; doi:10.1186/s40560-020-00516-6)
Supplement: Supplementary file 5 — Additional file 5: Table S3. Association between E/A and mortality at day-28 by logistic regression after adjustment for age. [file 40560_2020_516_MOESM5_ESM.docx]

| **Table S3** Association between E/A and mortality at day-28 by logistic regression after adjustment for age. | | | |
| --- | --- | --- | --- |
|  | **OR** | **CI95%** | ***P* value** |
| Age | 1.07 | 1.01-1.13 | 0.02 |
| E/A ratio at mitral valve | 0.22 | 0.03-1.13 | 0.07 |
| Survival was assessed at day-28. OR: odds ratio, CI95%: Confidence interval at 95%, E/A: ratio of early to late pulsed-wave Doppler of diastolic transmitral flow velocity. | | | |
